# Much More than a Cardiotonic Steroid: Modulation of Inflammation by Ouabain

**DOI:** 10.3389/fphys.2017.00895

**Published:** 2017-11-10

**Authors:** Luiz H. A. Cavalcante-Silva, Éssia de Almeida Lima, Deyse C. M. Carvalho, José M. de Sales-Neto, Anne K. de Abreu Alves, José G. F. M. Galvão, Juliane S. de França da Silva, Sandra Rodrigues-Mascarenhas

**Affiliations:** ^1^Programa de Pós-Graduação em Produtos Naturais e Sintéticos Bioativos, Laboratório de Imunobiotecnologia, Centro de Ciências da Saúde, Universidade Federal da Paraíba, João Pessoa, Brazil; ^2^Programa de Pós-Graduação em Biotecnologia, Laboratório de Imunobiotecnologia, Centro de Biotecnologia, Universidade Federal da Paraíba, João Pessoa, Brazil; ^3^Programa Multicêntrico de Pós-graduação em Ciências Fisiológicas, Laboratório de Imunobiotecnologia, Centro de Biotecnologia, Universidade Federal da Paraíba, João Pessoa, Brazil

**Keywords:** ouabain, immune system, peripheral inflammation, cell migration, cytokines, neuroinflammation

## Abstract

Since the discovery of ouabain as a cardiotonic steroid hormone present in higher mammals, research about it has progressed rapidly and several of its physiological and pharmacological effects have been described. Ouabain can behave as a stress hormone and adrenal cortex is its main source. Direct effects of ouabain are originated due to the binding to its receptor, the Na^+^/K^+^-ATPase, on target cells. This interaction can promote Na^+^ transport blockade or even activation of signaling transduction pathways (e.g., EGFR/Src-Ras-ERK pathway activation), independent of ion transport. Besides the well-known effect of ouabain on the cardiovascular system and blood pressure control, compelling evidence indicates that ouabain regulates a number of immune functions. Inflammation is a tightly coordinated immunological function that is also affected by ouabain. Indeed, this hormone can modulate many inflammatory events such as cell migration, vascular permeability, and cytokine production. Moreover, ouabain also interferes on neuroinflammation. However, it is not clear how ouabain controls these events. In this brief review, we summarize the updates of ouabain effect on several aspects of peripheral and central inflammation, bringing new insights into ouabain functions on the immune system.

## Introduction

Although the cardiotonic steroid ouabain was originally identified as a plant secondary metabolite (e.g., from *Strophantus gratus* and *Acokanthera ouabaio*), it was later described as an endogenous mammalian substance (Hamlyn et al., 1991) such as other cardiotonic steroids (e.g., marinobufagenin and digoxin) (Bagrov et al., 2009). Ouabain was found in bovine adrenal gland (Laredo et al., 1994; Schneider et al., 1998), adrenal gland tumors (Blanco and Wallace, 2013), bovine hypothalamus (Tymiak et al., 1993), bovine hypophysis (Schoner, 2002), and human plasma (Hamlyn et al., 1991; Ferrandi et al., 1997). It is noteworthy that ouabain isolated from mammalian tissues and body fluids is structurally, biochemically, and immunologically indistinguishable to ouabain isolated from plants (Schoner, 2002).

Ouabain synthesis seems to occur in the zona glomerulosa and fasciculata of the adrenal gland cortex (Masugi et al., 1988; Laredo et al., 1995), using hydroxycholesterol, pregnenolone, and progesterone (Hamlyn et al., 1998; Schoner and Scheiner-Bobis, 2007) as precursors. Thereafter, ouabain is released into the circulation after stimulation by adrenocorticotropic hormone (Lewis et al., 2014), epinephrine (Schoner and Scheiner-Bobis, 2005), angiotensin II (Laredo et al., 1997), and α1-adrenergic receptor agonists (Schoner, 2002; Schoner and Scheiner-Bobis, 2007). In rats, the physiological role of ouabain is associated to vasculature tone control and natriuresis (Nesher et al., 2009).

Ouabain levels are increased in different conditions such as chronic renal insufficiency (Stella et al., 2008), chronic salt intake (Blanco and Wallace, 2013), congestive heart failure (Manunta et al., 2009, 2010), hypertension (Hauck and Frishman, 2012), pregnancy (Dvela-Levitt et al., 2015), and primary hyperaldosteronism (Rossi et al., 1995). This steroid is also associated with stress conditions, such as physical exercise (Antolovic et al., 2000; Bauer et al., 2005). In addition, high levels of ouabain are correlated with cortisol concentration (Berendes et al., 2003), which reinforces its role as a stress hormone.

Besides its role as a Na^+^/K^+^-ATPase (sodium pump) inhibitor (Lingrel, 2010), which is associated with cardiovascular effects (Hamlyn and Blaustein, 2013; Blaustein et al., 2016), ouabain, at low concentrations, triggers Na^+^/K^+^-ATPase-mediated signaling pathways (Xie and Askari, 2002; Xie and Cai, 2003). These relayed signals cascades include Src kinase, MAPK, and NF-κB activation, reactive oxygen species release and others (Saunders and Scheiner-Bobis, 2004; Aperia, 2007). Ouabain induces several biological regulatory effects, including cell proliferation, hypertrophy, apoptosis (Bagrov et al., 2009) resulting in different functional outcomes. Additionally, it has been demonstrated that ouabain modulates various immune system functions (Rodrigues-Mascarenhas et al., 2009), including inflammation. In this mini-review, we present the relationship between ouabain and inflammatory process.

## Ouabain and immune system

The immune system is a highly specialized network of lymphoid organs, cells, humoral factors, and cytokines, which acts in order to maintain homeostasis (Parkin and Cohen, 2001). The relationship between ouabain and immune system was first studied when Quastel and Kaplan (1968) demonstrated that this steroid inhibits lymphocytes proliferation induced by the mitogen phytohaemagglutinin. This effect was later confirmed by several other reports that used different stimuli (e.g., anti-CD3 and IL-2) (Jensen et al., 1977; Dornand et al., 1986; Redondo et al., 1986; Olej et al., 1994; Brodie et al., 1995; Szamel et al., 1995). This phenomenon could be related to CD25 (Pires et al., 1997) and IL-2 reduced expression (Dornand et al., 1986; Szamel et al., 1995) induced by ouabain, since both molecules are required for lymphocyte proliferation. Moreover, ouabain reduces regulatory T cells absolute number in mice (Silva et al., 2015). It is noteworthy that CD25 is highly expressed and fundamental to regulatory T cells survival (Setiady et al., 2010). Besides that, it was also reported that ouabain induces cell death in stimulated lymphocytes (Olej et al., 1998; Esteves et al., 2005; Panayiotidis et al., 2010).

In thymocytes, T lymphocyte precursors, ouabain is able to modulate different events such as intracellular calcium concentration increase (Echevarria-Lima et al., 2003). It was also observed that this effect is related to CD69 increased expression, a molecule associated with cell activation, induced by ouabain (Rodrigues-Mascarenhas et al., 2003). Additionally, ouabain induces *in vitro* intracellular free radicals accumulation and thymocytes death (Smolyaninova et al., 2013). *In vivo*, ouabain synergizes with hydrocortisone increasing T lymphocyte precursors death by apoptosis (Rodrigues-Mascarenhas et al., 2006), which reinforces its role as a stress-related hormone. Moreover, ouabain reduced NFAT expression and P-p38 levels, after concanavalin A stimulation (Rodrigues-Mascarenhas et al., 2008, 2009). This later data support the fact that ouabain modulates cell signaling (Xie and Askari, 2002).

Ouabain is also able to modulate *in vivo* B lymphocytes dynamics, decreasing mature B cells in the bone marrow, spleen and peripheral blood (de Paiva et al., 2011), although IgG and IgM levels were not affected by ouabain. On the other hand, there was an increase in B lymphocytes of mesenteric lymph node, probably by CD62L reduced and CXCR5 increased expression (da Silva et al., 2015).

Despite ouabain effects on B and T lymphocytes, natural killer (NK) cells seem to be resistant to ouabain. In fact, ouabain did not affect NK cell cytotoxic activity, in neither the absence nor presence of stimulatory agents (de Moraes et al., 1989). However, ouabain inhibits lymphokine-activated killer (LAK) cell generation induced by IL-2 (Olej et al., 1994).

Many lymphocytes functions rely on antigen presenting cells (APCs), in which dendritic cells (DC) have a highlighted role together with macrophages (Steinman, 2012). The influence of ouabain on DC was also described. Nascimento et al. (2014) demonstrated that ouabain modulates dendritic cells markers and IL-12 production during activation by TNF-α. Additionally, ouabain affects monocyte/macrophage activation (Sowa and Przewłocki, 1997; Teixeira and Rumjanek, 2014). Indeed, ouabain reduces CD14 expression, a molecule involved in foreign antigens recognition, in human monocytes (Valente et al., 2009; Teixeira and Rumjanek, 2014). Moreover, ouabain inhibits a proinflammatory monocyte subset (mCD14^+^CD16^+^) appearance *in vitro*, which may indicate that this steroid also modulates the inflammatory response.

## Ouabain as a modulator of inflammation

Inflammation is an immunological complex response that can be triggered by pathogen- and damage-associated molecular patterns and is able to restore tissue homeostasis (Medzhitov, 2010). Acute inflammatory process is mainly characterized by vascular (e.g., vasodilation and vascular permeability) and cellular (e.g., leukocytes migration) alterations, resulting in five cardinal (clinical) signs: redness, swelling, heat, pain, and disturbance of function. Uncontrolled or unresolved inflammation can lead to homeostatic imbalance and chronic diseases, including cardiovascular and neurodegenerative diseases (Scrivo et al., 2011). Besides immune system role in inflammation, other systems, such as endocrine and nervous system, can also regulate this physiological response (Padro and Sanders, 2014; Procaccini et al., 2014). In fact, many hormones are known to affect inflammation, such as glucocorticoids (Cain and Cidlowski, 2017), and ghrelin, a pituitary-derived hormone (Baatar et al., 2011). In the following topics, ouabain role in the inflammatory process will be discussed.

### Ouabain and peripheral inflammation

Acute peripheral inflammation initiates after inflammatory signals recognition (e.g., infection and tissue injury) by resident cells, such as mast cells and macrophages. This recognition promotes mediators release (e.g., vasoactive amines and prostaglandins), which stimulates rapid effects on the vasculature, including vasodilation and fluid extravasation (i.e., increased vascular permeability; Medzhitov, 2008). One of the first reports associating ouabain and inflammation revealed that this steroid suppresses vascular permeability in the sheep skin and pleural cavity induced by the irritant agent turpentine (Lancaster and Vegad, 1967). Later, our group demonstrated that ouabain given intraperitoneally decreases zymosan-induced plasma extravasation in mice peritoneal cavity (Leite et al., 2015) and reduces the mouse paw edema stimulated by several phlogistic agents (de Vasconcelos et al., 2011). However, Gonçalves-de-Albuquerque et al. (2014) showed that intratracheal administration of ouabain induces lung edema formation in mice. It is important to consider that ouabain effect on lung edema must be related to Na^+^/K^+^-ATPase inhibition in alveolar cells (Gonçalves-de-Albuquerque et al., 2014), while ouabain effects demonstrated by our group may be associated with cell signaling mechanisms in immune cell (e.g., P-p38 and NF-κB activity inhibition; Mascarenhas et al., 2014; Leite et al., 2015).

Vasodilation and vascular permeability are events tune regulated by vasoactive amines. Histamine, which plays a critical role among these vasoactive molecules, is released by perivascular mast cells together with other mediators (e.g., newly synthesized cytokines and tryptases) during inflammation (Nathan, 2002). Different ouabain effects on histamine secretion by mast cells have been described. Okazaki et al. (1976) reported that ouabain inhibits antigen-induced histamine release on guinea-pig mast cells. On the other hand, several studies revealed that ouabain increased histamine secretion induced by different agents on rat mast cells (Frossard et al., 1983; Amellal et al., 1984; Knudsen et al., 1992; Lago et al., 2001), while no ouabain effect on human mast cells (Senol et al., 2007) and basophils (Magro, 1977) were reported. Different protocols and species variation in the ouabain sensitivity of Na^+^/K^+^-ATPase (Abeywardena et al., 1984; Herzig and Mohr, 1984; Wang et al., 2001) could explain this discrepant ouabain effects on mast cell degranulation.

Upon initiation of acute inflammation, circulating leukocytes are able to recognize molecules (e.g., selectins, integrins, and chemokines) on activated vascular endothelium and, after rolling and adhesion steps, they cross blood vessel barrier and reach inflamed tissue (Ley et al., 2007; Vestweber, 2015; Kourtzelis et al., 2017). Neutrophils are the first cells recruited to the injured site, followed by other inflammatory cells such as monocytes (Kolaczkowska and Kubes, 2013; Wang and Arase, 2014). These polymorphonuclear leukocytes are important not only to eliminate microorganisms but they also play a key role in inflammation resolution (Mayadas et al., 2014; Sugimoto et al., 2016). Moreover, neutrophils role in chronic inflammation has been described and they are pointed as a target to emerging therapeutic strategies (Soehnlein et al., 2017). Considering this, blocking neutrophil recruitment appears to be a crucial way to avoid inflammation maintenance.

Ward and Becker (1970) initially described ouabain inhibitory effect on rabbit neutrophil migration toward bacterial chemotactic factors *in vitro*. In agreement with this study, our group revealed that ouabain pretreatment reduces mice neutrophil migration induced by zymosan, a component of the cell wall of yeast *Saccharomyces cerevisiae*, (Leite et al., 2015) and by *Leishmania amazonensis* (Jacob et al., 2013). These data provide clear evidence that ouabain inhibits neutrophil recruitment induced by microbial agents. This ouabain effect was also demonstrated in peritoneal inflammation induced by mitogen concanavalin A (de Vasconcelos et al., 2011). Furthermore, in airway allergic inflammation model, ouabain has an anti-migratory effect (Galvão et al., 2017). Ray and Samanta (1997) have also demonstrated that ouabain impairs *in vitro* human neutrophil migration, by interfering with IL-8 receptor recycling. Additionally, other studies have demonstrated that ouabain also decreases lung cancer cells migration (Liu et al., 2013), possibly by reducing the expression of molecules related to cell adhesion (e.g., integrins and ICAM) (Takada et al., 2009; Ninsontia and Chanvorachote, 2014) and cell migration (e.g., Src, Akt, and FAK) (Pongrakhananon et al., 2013; Shin et al., 2015).

However, the inhibitory effect of ouabain on cell migration seems to depend on the presence of a previous inflammatory stimulus, since ouabain itself given by inhalation (Feng et al., 2011) or intratracheally (Gonçalves-de-Albuquerque et al., 2014) causes acute lung inflammation with increased neutrophil migration. This proinflammatory effect was followed by LTB_4_ and PGE_2_ high levels, both lipid mediators associated with cell migration. Moreover, it has been demonstrated that ouabain at high concentrations induces VCAM-1 expression (an adhesion molecule) in murine endothelial cells (Bereta et al., 1995). Na^+^/K^+^-ATPase inhibition may be, at least partially, responsible for this ouabain effect (Lacroix-Lamandé et al., 2012; Gonçalves-de-Albuquerque et al., 2014), but Na^+^/K^+^-ATPase-dependent activation of signaling cascades (e.g., ERK and p38 MAPK) cannot be ruled out (Bereta et al., 1995; Feng et al., 2011). Indeed, Leu et al. (1973) demonstrated that ouabain stimulates guinea-pig alveolar and peritoneal macrophages migration independent of the sodium pump.

A different pattern of cytokines is present since the inflammation onset until resolution phase. These soluble proteins are secreted by a variety of cells (e.g., immune e non-immune cells) and allow intercellular communication, mediating and regulating inflammatory process. An imbalance in proinflammatory (e.g., TNF-α) and anti-inflammatory (e.g., IL-10) cytokine production could entail inflammatory disorders (Tayal and Kalra, 2008; Sugimoto et al., 2016). Monocytes/macrophages are immune cells that act as a key source of cytokines because of their plasticity ability (i.e., change their pattern of cytokines and functions when exposed to different signals; Gordon and Taylor, 2005; Mantovani et al., 2014). It has been described that ouabain itself can stimulate human monocytes to secrete cytokines such as IL-1α, IL-1β, IL-6, and TNF-α (Foey et al., 1997; Matsumori et al., 1997, 2000; Teixeira and Rumjanek, 2014). Some different results are related to IL-6 and TNF-α production, which could be associated with individual variability of human donors. Indeed, critically ill patients with high levels of ouabain had higher serum concentrations of these proinflammatory cytokines and other inflammatory markers, such as C-reactive peptide and serum amyloid A, when compared to patients with low ouabain concentrations (Berendes et al., 2003). Interestingly, ouabain enhances interleukin-10 levels in human monocytes (Teixeira and Rumjanek, 2014). Recently, Kobayashi et al. (2017) demonstrated that ouabain effect on IL-1β release, in both macrophages and cardiac tissue, is related to NLRP3 inflammasome activation, which in turn is mediated through K^+^ efflux. It is noteworthy that in this study the authors used high doses of ouabain both *in vitro* and *in vivo*. This contrasts with other studies that show a reduction of a different pattern of cytokines, including IL-1β (Leite et al., 2015) with lower ouabain doses in presence of inflammatory stimulus (Jacob et al., 2013; Galvão et al., 2017).

Despite the well-established proinflammatory activities of some cytokines, such as TNF-α and IL-6, some studies have demonstrated their anti-inflammatory role (Liu et al., 1998; Zakharova and Ziegler, 2005; Masli and Turpie, 2008; Scheller et al., 2013). In this regard, use low concentrations of ouabain as cytokine immunoregulator could be useful in different clinical conditions. In fact, ouabain at low doses reverses sepsis-induced immunoparalysis by increase TNF-α, IFN-γ, and GM-CSF levels and improve mice survival (Dan et al., 2014).

Pain is another cardinal sign of inflammation, which is also modulated by ouabain. de Vasconcelos et al. (2011) demonstrated that intraperitoneal administration of ouabain reduces nociceptive behavior in mice model of inflammatory pain (i.e., acetic acid induced writhing test). This steroid also induces supraspinal antinociceptive activity, related to opioid mechanisms, since naloxone, an opioid antagonist, inhibits its effect. In addition, other studies revealed that ouabain intracerebroventricular (i.c.v.) (Calcutt et al., 1971) and intratechal (i.t.) (Zeng et al., 1999) injections at relative high doses (micrograms) produce central antinociception and potentiate morphine and clonidine central antinociceptive effect, mainly by enhancement of cholinergic transmission at the spinal cord level (Zeng et al., 1999, 2007). In contrast, it has also been shown that low doses (nanograms) of ouabain (i.c.v.) antagonize opioid receptor agonists (Masocha et al., 2003, 2016; Gonzalez et al., 2012) and that ouabain (i.t.) itself did not cause antinociception (Horvath et al., 2003). This pain modulation by ouabain, which depends on the dose and administration route used, suggests that it can modulate events in the central nervous system such as neuroinflammation.

### Ouabain and neuroinflammation

Some studies have described ouabain role in the central nervous system (CNS). However, the effects of this steroid on neuroinflammation can be controversial. In a study with rat hippocampus, ouabain anti-inflammatory effect against neuroinflammation induced by LPS was observed. Acute intraperitoneal pre-treatment with this steroid reduced iNOS and IL-1β mRNA levels. In addition, ouabain also reduced p65 subunit NF-κB translocation and IκB degradation, both mechanisms important to inflammatory process (Kinoshita et al., 2014). However, when ouabain is administrated by intrahippocampal route in a concentration that does not inhibit Na^+^/K^+^-ATPase, it induces activation of NF-κB and increases iNOS and TNF-α mRNA levels (Kawamoto et al., 2012). As well as in peripheral inflammation, the anti-inflammatory effect of ouabain on hippocampus was only observed after an inflammatory stimulus. This could explain the different effects regarding NF-κB activation. Additionally, another study demonstrated the ability of ouabain to restore the lipid composition of rat hippocampal membranes in neuroinflammation induced by LPS (Garcia et al., 2015).

In rat cerebellar cell culture, ouabain at high concentrations induced NF-κB activation and consequent TNF-α and IL-1β cytokines increase through NMDA-Src-Ras pathway in absence of inflammatory stimulus (de Sá Lima et al., 2013). However, ouabain decreases IL-1β release in LPS-stimulated astrocytes (Forshammar et al., 2011). In spite of that, ouabain did not modulate IL-1β release in LPS-stimulated microglia, while increased TNF-α release at low concentration (Forshammar et al., 2013). Therefore, ouabain role in cytokine production at CNS level depends on cell type and concentration used. Besides that, ouabain effects on CNS could be associated with its role in bipolar and depressive disorders (Goldstein et al., 2006; Tonin et al., 2014). The interaction between neuroinflammation and cardiac steroids is more substantially detailed by Orellana et al. (2016).

## Conclusions and perspectives

**Figure 1:**
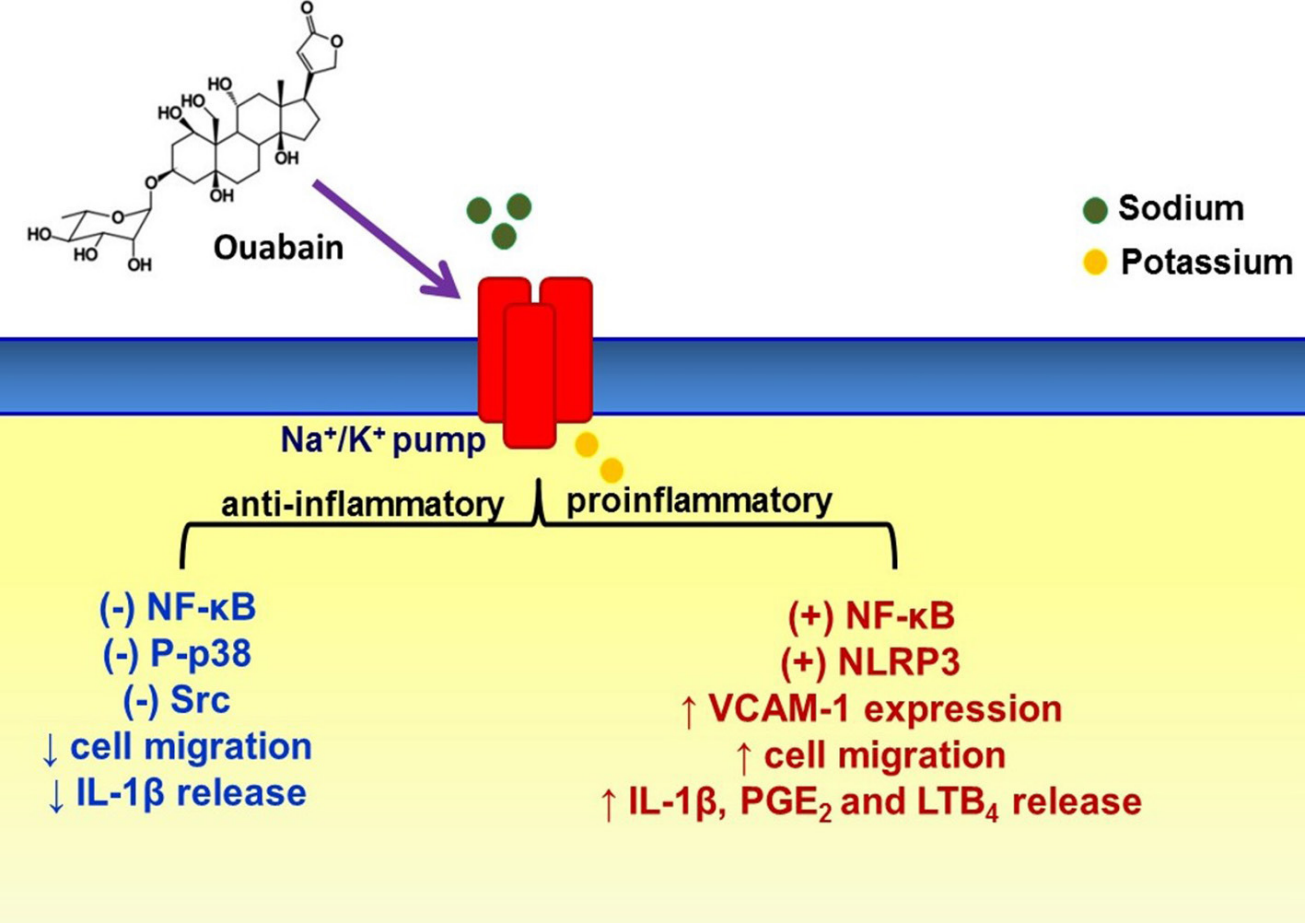
Some cellular and molecular events modulated by ouabain on inflammatory process. Dual (pro- and anti-inflammatory) ouabain effects on the inflammation depend on some conditions such as ouabain concentration, cell type, and even functional state. This steroid hormone can modulate some cell signaling pathways due to Na^+^/K^+^-pump binding, which not necessarily means pump inhibition. (+) activation, (–) inhibition, ↑ increase, ↓ decrease.

In summary, compelling evidence indicates that ouabain has a pro- and anti-inflammatory effects (Figure 1), which mainly depends on its concentration and functional state of cells (i.e., absence or presence of inflammatory stimulus), corroborating other ouabain effects on the immune system. However, to the best of our knowledge, studies relating ouabain and chronic inflammation are missing. In addition, more details about ouabain mechanism of action are necessary. Lastly, ouabain effects on the inflammatory process could be better explored in order to establish possible strategies for pharmacological treatment of immune dysregulation/inflammatory diseases.

## Author contributions

Conceived and designed the manuscript: SR-M and LC-S. Wrote the manuscript: LC-S, AAA, DC, ÉAL, JG, JMS-N, and JFS. Final version: SR-M.

### Conflict of interest statement

The authors declare that the research was conducted in the absence of any commercial or financial relationships that could be construed as a potential conflict of interest.
