# Corrigendum: Much More than a Cardiotonic Steroid: Modulation of Inflammation by Ouabain

**DOI:** 10.3389/fphys.2018.00001

**Published:** 2018-01-12

**Authors:** Luiz H. A. Cavalcante-Silva, Éssia de Almeida Lima, Deyse C. M. Carvalho, José M. de Sales-Neto, Anne K. de Abreu Alves, José G. F. M. Galvão, Juliane S. de França da Silva, Sandra Rodrigues-Mascarenhas

**Affiliations:** ^1^Programa de Pós-Graduação em Produtos Naturais e Sintéticos Bioativos, Laboratório de Imunobiotecnologia, Centro de Ciências da Saúde, Universidade Federal da Paraíba, João Pessoa, Brazil; ^2^Programa de Pós-Graduação em Biotecnologia, Laboratório de Imunobiotecnologia, Centro de Biotecnologia, Universidade Federal da Paraíba, João Pessoa, Brazil; ^3^Programa Multicêntrico de Pós-graduação em Ciências Fisiológicas, Laboratório de Imunobiotecnologia, Centro de Biotecnologia, Universidade Federal da Paraíba, João Pessoa, Brazil

**Keywords:** ouabain, immune system, peripheral inflammation, cell migration, cytokines, neuroinflammation

In the original article, we neglected to include the funder CAPES/PROCAD-2013, grant number 2951/2014. The authors apologize for this error and state that this does not change the scientific conclusions of the article in any way.

## Conflict of interest statement

The authors declare that the research was conducted in the absence of any commercial or financial relationships that could be construed as a potential conflict of interest.

